# Assessment of Prevalence and Risk Factors for Intestinal Parasitosis, Malnutrition, and Anemia among School Children in Ghindae Area, Eritrea

**DOI:** 10.1155/2020/4230260

**Published:** 2020-10-29

**Authors:** Yafet Kesete, Huruy Tesfahiwet, Ghimja Fessehaye, Yohana Kidane, Yafet Tekle, Asmerom Yacob, Biemnet Seltene

**Affiliations:** ^1^Department of Clinical Laboratory Science, Asmara College of Health Sciences, Asmara, Eritrea; ^2^Nakfa Hospital, Nakfa, Eritrea; ^3^Gonie Community Hospital, Barentu, Eritrea; ^4^Department of Microbiology, Eritrea Institute of Technology, Mai Nefhi, Eritrea; ^5^Sembel Referral Hospital, Asmara, Eritrea; ^6^Massawa Zonal Hospital, Massawa, Eritrea; ^7^Ghedem Hospital, Ghedem, Eritrea; ^8^Godaif Community Hospital, Asmara, Eritrea

## Abstract

**Background:**

Research studies on determination of risk factors for intestinal parasitic infections and related malnutrition and anemia in various tropical areas are necessary for appropriate preventive resource allocation and cost effective control. This study is aimed at evaluating the prevalence and risk factors of intestinal parasitosis, malnutrition, and anemia amongst elementary and junior school students in Ghindae area, Eritrea.

**Method:**

A cross-sectional study was conducted in 6 schools around Ghindae from February to April 2018. 460 children were selected randomly for analysis and consent was taken from guardians. The pertinent sociodemographic data was collected using a pretested questionnaire, and anthropometric measurements were carried out to determine the proportion of students with malnutrition, stunting, and thinness. Fecal samples were examined by formal-ether concentration technique, and blood specimen was collected and analyzed for the assessment of hemoglobin using Hemocue analyzers. The association between predictors and outcome variables were measured with a stepwise logistic regression model.

**Result:**

The overall prevalence of intestinal parasitosis was 45.3%. Protozoan infections (38.2%) were more prevalent than soil-transmitted helminthic infections (10.4%). The presence of different intestinal parasitic infections had statistically significant association with the residence area, hand washing habits, source of drinking water, and type of latrine used. The prevalence of malnutrition was 36.9% with 18.5% stunting and 21.2% thinness. Students of rural areas had 2.03 times more odds of having malnutrition. The current prevalence of anemia was 12.4%, out of which 7.6% had mild anemia while 4.4% of them had moderate anemia and 0.4% were severely anemic.

**Conclusion:**

The prevalence of parasitic infection among school-age children in Ghindae area was high. Unsafe drinking water sources from streams and springs were among the core problems for increased prevalence along with decreased awareness on personal hygienic and sanitation practices. Undernutrition was widely prevalent among students in Ghindae area. Height for age and BMI for age scores of all participants were found to be below the WHO growth standards. Malnutrition was in higher prevalence in rural areas outside Ghindae, which is attributed to two times higher odds than their counterparts. The magnitude of anemia found in this study is considered a mild public health problem.

## 1. Background

In this modern era, intestinal parasitic infections (IPIs), malnutrition, and anemia still continue to become the most common public health issues affecting school children especially in underdeveloped countries including Eritrea [1, 2]. More than a billion and half people are infected with soil-transmitted helminths (STH) globally in which over 568 million school-age children and above 267 million preschool children reside in helminthiasis prevalent areas [1]. Currently, 1 in 9 people (820 million) worldwide are hungry or undernourished where more than 90% of the world's stunted children reside in Africa and Asia with numbers rising since 2015 [3, 4]. World Health Organization estimated that about 40% of the world's population or more than 2 billion people suffer from anemia. In rural areas of developing countries, these are the major causes of morbidity in primary and junior school students who have the greatest prevalence of worm infestation, malnourishment, and anemia [5].

These three medical conditions are highly interrelated and usually present in individuals simultaneously. Distributions of IPIs are closely linked to lack of sanitation, shortage of access to safe water, and proper hygiene practices; therefore, they are common whenever there is poverty [6, 7]. IPIs directly affect the survival, growth, appetite, physical fitness, and scholastic performance of children and gradually aggravate the nutritional status and increase morbidity of children as they become more at risk for infectious diseases like tuberculosis and pneumonia [1, 8].

Parasitic infections like helminthiasis cause malnutrition through different mechanisms which include increasing metabolic requirements, decreasing food intake, excessive nutrient absorption, and direct loss of nutrients [9]. Presence of malnutrition during childhood has a key significance in adult health, work productivity, and economic achievement of developing countries [10]. The relationship between intestinal parasitosis and anemia, particularly microcytic hypochromic anemia and iron deficiency, is also well established. This depends on the major invasive parasites especially hookworm infection, schistosomiasis, and amoebiasis on which iron deficiency anemia occurs especially in children of low income families due to high demand of iron during a period of fast growth [11].

To be effective, interventions aimed at reducing the effects of parasitic infection, malnutrition, and anemia need to be based on a proper assessment of the current situation. No adequate previous studies have been conducted on the assessments of intestinal parasitosis, malnutrition, and anemia in Eritrea which could have been used as reference. Those which have been published are very few. Therefore, the study can be used as baseline data for better control and prevention strategies. This study is aimed at evaluating the prevalence and risk factors of intestinal parasitosis, malnutrition, and related anemia amongst elementary and junior school students in Ghindae area, northern Red Sea region, Eritrea. This study will identify the high risk population that is fundamental for appropriate resource allocation, reliable estimation of the overall drug needs of programs, and efficient implementation of preventive measures.

## 2. Methods

### 2.1. Study Design and Study Population

A cross-sectional study was conducted from February 2018 up to April 2018 in Ghindae town, northern Red Sea region, Eritrea. The town is located at an altitude of 1020 meters above sea level (masl) with 15° 26′ N 39° 05′ E and at about 48 km from Asmara, the capital of Eritrea. The town along with two other smaller towns, Embatkala and Dongolo, is comprised of one administrative subzone in which around 12,000 families and approximately 54,000 people live within its boundary. Ghindae domain entertains three climates at different seasons: cooler highland climate, semihot lowlands, and lowland climate. Five years' average annual rainfall for that place was 800 mm with bimodal rainy seasons yielding an everlasting river that flows through the town predisposing the children to waterborne diseases. There is one regional referral hospital along with few healthcare centers. The total population of elementary and junior school students in Ghindae area is 7895 attending in 10 different schools with 3451 being females and 4444 being males.

### 2.2. Sampling Technique

**Figure 1:**
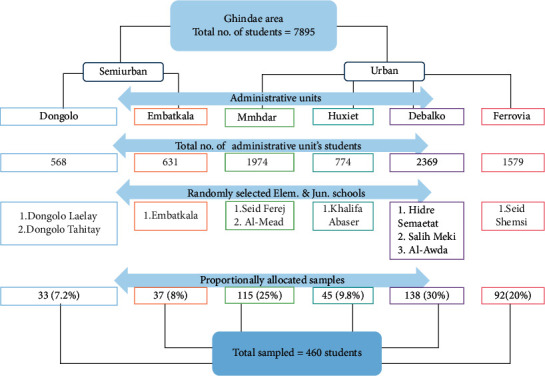
Schematic presentation of sampling procedure in administrative units of Ghindae town, 2018.

A sample size of 460 was required assuming a 50% prevalence for no other similar previous studies, margin of error of 5% and a 20% contingency for nonresponse. A disproportionate random stratified sampling was utilized to select subjects from schools. Ghindae area is organized into 6 administrative units containing 10 public elementary and junior (E.J) schools, all owned by the government. Each school serves students from specifically assigned administrative unit. For this research, one school was selected randomly from each administrative unit. The proportion of students from each school was then calculated directly out of total proportion of students from each administrative unit (Figure 1). To select the sample children, the students were first stratified according to their educational level (grade 1 to grade 8). A quota was then allocated for each grade and each classroom. Finally, the participant students were selected using systematic random sampling techniques by using class registers as the sample frame.

### 2.3. Sociodemographic Data Collection

The pertinent data for the study were collected using a pretested, precoded questionnaire. Participants were interviewed by research members to assesss the baseline sociodemographic data as well as health status data, including previous or current medical risk factors that are known to expose to intestinal parasitosis, malnutrition, and anemia. The questionnaire was prepared for guardians and students individually. Most of the sociodemographic data was collected by interviewing the parents or guardians. Observational exam was also performed to study some variables, like hand hygiene and cleanliness of fingernails. Physical cleanliness of hands was evaluated by checking the finger pads, fingertips, palm, and the back of both hands. Hands were regarded as unclean if any noticeable dirt was seen and clean if there was no visible dirt. To assess finger nail hygiene of both hands, trimmed fingers were considered as clean and untrimmed nails with accumulated dirt were considered as unclean.

### 2.4. Anthropometry

Anthropometric data like age, height, and weight of the study participants were collected at school compounds from February 10 to April 30, 2018. A stadiometer with a sliding headpiece was used to record student's height barefooted. Each student was also weighed with minimum clothing using a portable weight scale which was calibrated daily. Anthropometric measurements were converted into height for age (HAZ) and body mass index (BMI) for age (BAZ) Z-scores using WHO growth reference [12]. Students which were below −2 Z-score for weight for age, body mass index for age, and height for, age were regarded as underweight, thin, and stunted, respectively.

### 2.5. Stool Specimen Collection and Analysis

Each participant was provided with a prelabelled, clean, wide-mouthed 20 ml stool container with screw caps instructing the guardians to use the spatula attached to the cover of the bottle to collect fresh stool sample from their children the next morning. Subjects were advised on proper handling of stool samples and providing early morning fecal samples which usually present with more parasite than those collected at other times [13]. All teachers were requested humbly in sample collection for full cooperation.

About thumb size (10 g) fresh stool specimens were collected from each subject. Each sample was fixed in 10% formalin immediately after collection and examined at Asmara College of Health Science parasitology laboratory within 24 hours after collection. Stool samples were examined using standard procedure of formal-ether concentration technique [14]. From the emulsified sample, 1 g (thumb size) of feces was added to about 4 ml of 10% formalin and then mixed and sieved in another tube. Then 3-4 ml of diethyl ether was added and centrifuged immediately at 750–1000 g (∼3000 rpm) for 1 min. Finally the supernatant was discarded, and then small portion of the sediment was transferred to a slide and covered with cover slip and examined first with 10x and then 40x objectives and also iodine stained slides were prepared and examined microscopically.

### 2.6. Blood Specimen Collection and Analysis

Hemoglobin level of participants was determined using Hemocue model 201 analyzer measuring finger-prick blood sample in the school compound. A short training on the machine operation was given to research members before the actual sample collection period.

Measured hemoglobin level was divided according to severity into four groups for two age categories based on WHO standards [15]. For children aged 6 to 11 years, above 11.5 g/dL is normal, 11.0–11.4 g/dL mild anemia, 8.0–10.9 g/dL moderate, and <8.0 g/dL severe anemia. For children aged 12 to 14 years, above 12 g/dL is normal, 11.0–11.9 g/dL mild anemia, 7.0–10.9 g/dL moderate, and <7.0 g/dL severe anemia. Hemoglobin readings were adjusted for altitude based on WHO standard [15].

### 2.7. Quality Control

Content and face validity of English and then Tigrigna (local language) version of the questionnaire was determined through supervision of experts in the field of clinical laboratory, public health, and environmental health. Data and sample collectors were fourth-year clinical laboratory science students which were trained to ascertain a common understanding by employing thorough discussion sessions and in-house practice programs using role play interviews. Thereafter, pretesting was conducted in the field prior to actual data collection period on 42 subjects.

For accurate measurements of weight and height, the portable weight scale was calibrated daily using standard calibration weights of 2 kg iron bars. The weight and height measurement of each participant was done twice and the average was recorded. Also the Hemocue blood analyzers were checked on a daily basis using the reference microcuvettes as indicated by the manufacturer.

### 2.8. Inclusion and Exclusion Criteria

Students who reside in Ghindae area and enrolled in the preselected schools during the study period were included in the study. Students who have taken anthelminthic medication within two weeks before study [13] or who were not able to submit blood or stool samples and specimens contaminated by urine, water , or other materials were excluded from the study.

### 2.9. Statistical Analysis

The data was analyzed using SPSS statistical software version 20. Descriptive statistics was used to evaluate the data. Differences in the prevalence and intensity of infection between age and sex were tested using the Pearson chi-square/Fisher's exact tests. One-way ANOVA and independent *t*-tests were used to analyze hematological parameters. Kernel density plot was used to express the proportion of anthropometric outcomes. Bivariate and stepwise logistic regression model analysis were carried out to assess the association between independent and outcome variables. A 5% significant level was taken as a minimum level of significance. Output data was presented using tables and figures.

### 2.10. Ethical Considerations

Ethical clearance was acquired from the Asmara College of Health Science research ethical committee and Ministry of Health (MOH). The researchers obtained verbal and written consent from guardians of students on the consent form attached with questionnaire, and it remained anonymous. Capillary blood collection was performed after obtaining a signed written informed consent from parents and an oral assent from participants. Only student code number was used to retrieve the parasitological, anthropometric, and hemoglobin results and was held confidential. Report of positive individuals was notified to concerned parties for proper treatment according to standard guidelines.

## 3. Results

### 3.1. Sociodemographic Characteristics of the Study Participants

From the 460 students selected for the research, 450 children were present with fully completed questionnaire and anthropometric measurement and were able to provide appropriate stool and blood specimens with a response rate of 97.6%. The study included school children of age ranging between 6 and 16 years out of which 215 (47.8%) were males and the mean (±SD) age was 10.34 (SD ± 2.6) years. As to the income, 86 (24.4%) of the house heads earned a mean monthly income of below 500 Nakfa (US$1 = 15 Eritrean Nakfa). Regarding the educational status of the parents of the sampled children, 49 (10.9%) of the fathers had no formal education; 141 (31.3%) had attended secondary school; 88 (19.6%) mothers of the children were illiterate. As to the occupation of the parents of the children, majority of fathers, 144 (32%), were part of national service program and 354 (78.7%) mothers were housewives.

### 3.2. Prevalence Outcomes of Parasitosis, Malnutrition, and Anemia

The result of the stool specimen analysis showed that 204 (45.3%) (95% CI = 40.9–49.8) children tested positive for one or more intestinal parasitic infection (Table 1). Four intestinal parasite species, i.e., *Entamoeba histolytica/dispar*, *Giardia duodenalis*, *Hymenolepis nana*, and hookworm, were identified. The combination of *E. histolytica/dispar* and *G. duodenalis* was the most predominant in double infection, accounting for 65.2% of the total infected study subjects. The prevalence of different intestinal parasites was also variable among the six administrative units around Ghindae (Table 2).

**Figure 2:**
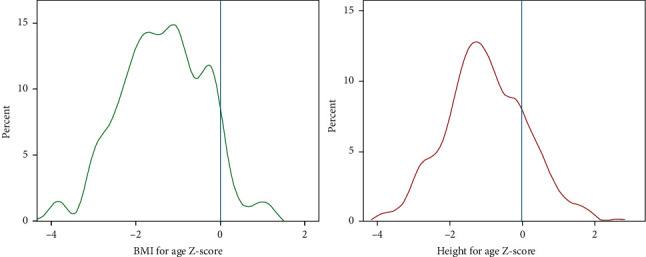
Kernel density plots of BMI for age and height for age Z-scores for all students in Ghindae area, Eritrea, 2018.

The overall prevalence of malnutrition was 36.9% (95% CI = 33.0–41.7). Out of the studied school children, 18.5% were stunted (HAZ < −2SD) and 21.2% were wasted (BAZ < −2SD). Also severe stunting (HAZ < −3SD) and thinness (BAZ < −3SD) was observed with prevalence of 3.6% and 4.9%, respectively. Generally, Z-scores of height for age and BMI for age for all students were found to be below the 2006 WHO standards (Figure 2).

After blood specimen analysis, the study population was divided into anemic and nonanemic groups. The anemic group was further categorized into mild, moderate, and severe anemia. Prevalence of anemia among primary school children was 12.4% (95% CI = 9.6–15.6). Upon further classification, 7.6% of the anemic group were mildly anemic and the remaining 4.4% and 0.4% were moderately and severely anemic, respectively. The mean ± SD hemoglobin was 12.85 ± 1.19 g/dl. The mean ± SD value of hemoglobin in males was 12.92 ± 1.26 g/dl and 12.78 ± 1.13 g/dl in females.

### 3.3. Correlates of Parasitosis, Malnutrition, and Anemia

As indicated in Table 3, the bivariate analysis showed that ethnicity, maternal age, hand cleanliness, awareness of purpose of hand washing, source of drinking water, and having history of bloody diarrhea were significantly associated with protozoan and helminthic intestinal infections. Children with age group 6–11 were present with more *G. duodenalis* and *H. nana* infection than those aged 12–16 (*p* < 0.05). In addition, the Saho ethnic group had a statistically significant higher rate of protozoan infection compared to other ethnic groups (COR = 1.77, 95% CI = 1.07–2.93).

Double infection was in lower prevalence in families who have their own latrine (COR = 0.40, 95% CI = 0.19–0.84). Students whose families use a common latrine along with other families had a significantly higher prevalence of giardiasis (AOR = 2.07, 95% CI = 1.20–3.59) than those who had their private latrine. A statistically significant presence of giardiasis was also observed in students who had been treated before for intestinal infection (*p*=0.005). Generally, helminthic infections were significantly higher in students whose mothers are aged below 24 and who had history of bloody diarrhea. The prevalence of hookworm infection was observed in statistically significant difference among urban and rural settlements of students. The prevalence was highest in Dongolo (12.5%), a rural area around Ghindae.

A multivariable analysis was performed for variables that showed statistically significant association with intestinal protozoan and helminthic infection at bivariate level. Source of drinking water, hand hygiene, and knowing the purpose of washing hands remained as the independent predictors among the studied children (Table 4). Households who use streams and river water as a source for drinking were 2.41 times at higher odds of having protozoan infection (AOR = 2.41, 95% CI = 1.04–5.58).

In bivariate logistic regression, age, residence area, type of school, grade, and waste disposal system were found to be significantly associated with malnutrition (Table 5). However, after stepwise logistic regression analysis, only residence of students had strong association with malnutrition. Students of rural areas like Embatkala and Dongolo had two times more odds of having malnutrition (AOR = 2.03, 95% CI = 1.04–3.96) than their counterparts (Table 6). The prevalence of malnutrition was not affected by parental educational status, ethnicity, and religion. There was no statistically significant association between anemia and thinness.

In this study, significant relationships were also observed between anemia and residence of students (*p* < 0.05). Adolescents aged 12–16 years had 1.36 times higher odds of being anemic (AOR = 1.36, 95% CI: 1.09, 3.42) compared to those aged 6 to 11 (Table 6). Female students had higher prevalence of moderate anemia than males (*p* < 0.05). Higher prevalence of hookworm infection was present in anemic students (40%), compared with 12.2% of anemic school children who had no hookworm (*p*=0.06).

## 4. Discussion

The study showed widespread prevalence of intestinal parasitosis among school children in Ghindae area. The overall prevalence of intestinal parasite (45.3%) was consistent with the studies conducted at different parts of Africa [16–18]. The present study, however, had higher prevalence of IPIs in comparison to similar research done in other parts of Eritrea [19].

Students whose source of household water supply was from river stream were 2.41 times more likely to have protozoan infection which is more associated with unsafe water sources. Most of the schools did not have functioning toilets contributing to the problem by practice of open field defecation at the nearby river. However, some meta-analytical studies on causes of diarrheal diseases have determined point use contamination as a practical factor in contagion of protozoan infections in some settings [20, 21]. Therefore, these diseases are not considered to be solely transmitted through water and as the river is used for small scale farming purposes, spread may also be through improperly washed and undercooked green vegetables. Also trade and economic activities are more at the center of town and students are exposed to street food which, upon sharing, can be part of the reason for the transmission dynamics.

Similar to previous reports [22, 23], results of the study showed that protozoan infections (38.2%) were more common compared with STH infections (10.4%). The high presence of double infection in Ghindae town that is mainly due to amoebiasis and giardiasis was probably attributed to the key source of drinking water, the local river, on which almost all of households dispose their household and human wastes. Moreover, the climate is warm and is highly humid which directly relates to high consumption of water among residents making it favorable for protozoan infection which is mainly waterborne disease.

Prevalence of *E. histolytica/dispar* (24.3%), the most frequently found parasite along with *G. duodenalis* (21.6%), was higher compared to other studies conducted elsewhere [24, 25]. The higher predominance of *G. duodenalis* and *E. histolytica/dispar* infection may also be on account of their capacity to withstand normal level of chlorine treatment in drinking water [26]. *H. nana* had a prevalence rate of 9.6% and was comparable to the report from the other studies [18, 24]. The Saho ethnic group had statistically significant higher rate of protozoan infection probably attributed to isolated living style in a peripheral vicinity in which sole source of drinking water is the river.

Compared to subjects without usage of latrines, lower prevalence rates of intestinal parasitosis were observed among private or public latrine users which was in line with other studies [17]. In consistence with other studies [17, 24], students who do not know the purpose of washing hands and with dirt in their fingernails had 2.23 and 1.89 more likelihood of having parasitic infection depicting poor hygiene practices.

Hookworm was significantly prevalent in Dongolo area, a rural periphery around Ghindae town, attributed to the lifestyle of families who are engaged in agricultural pursuits, associated with widespread use of human feces as soil fertilizer [27]. Moreover, the sandy-silt type of soil in that area is conducive for maturation of hookworm filariform larvae [27].

The overall prevalence of malnutrition in this study was 36.9% and the most frequent type of malnutrition was stunting (low BMI for age) (21.3%). This was higher than the previous prevalence estimated for the country (31.1%) [28]; however, the distribution was within the range reported for Africa [29] and in agreement with other studies [30]. High stunting could be due to a prolonged shortage of balanced meals, especially amongst children from poor families due to frequent erratic rainfall with low farm production leading to reduced food [12, 31]. The proportion of malnourished children was higher in the children aged 12–16 than that of 6–11 years consistent with another study in horn of Africa [32].

Thinness and malnutrition were in higher prevalence in semiurban areas outside Ghindae town in agreement with other studies [33]. Students living in rural areas like Embatkala and Dongolo had 2.03 times higher odds of having malnutrition than their counterparts. This supports the national poverty assessment denoting the high fraction of population (80%) who are unable to meet their essential food requirements in which 80% are in rural areas and 20% in urban areas [31].

Similar to other studies [30], students whose mothers' occupation is other than housewife had higher prevalence of malnutrition as the children will be overlooked after their dietary intake when their mothers spend their time away. Also students who have parents currently divorced had higher proportion of low BMI for age (thinness) on account of low socioeconomic status and income present in students with divorced parents. In agreement with previous studies [34], anthropometric scores were not associated with overall rate of intestinal parasitic infections (*p* > 0.05). The lack of association may be explained by the absence of measurable differences or due to very low parasitic load.

The magnitude of anemia determined in this study (12.4%) is considered a mild public health problem according to WHO standards [35]. The prevalence rates of mild, moderate, and severe (Hb < 7 g/dl) anemia were 7.6%, 4.4%, and 0.4%, respectively. Anemia prevalence in the study group was consistent with a recent prevalence report from neighboring countries [18] but lower than other studies around the world [36, 37] as determined by the same techniques. Adolescents (12–16 years) had higher prevalence of anemia (15.2%) than their counterpart in agreement with previous studies [18].

Maternal education did not show significant association with anemia in contrary to findings of several studies [38, 39] probably due to the homogeneity of the respondents' educational status as most of those who had no formal education were in the education for elders' program which targets elimination of illiteracy. Similar to other studies, increased number of family members was significantly associated with severity of anemia (*p*=0.001) [36]. Unlike other studies [36, 39], income of families was not observed to be related to anemia as estimate of family income was derived from salary of household head that does not include other income sources like remittance income present in significant part of sample size and inability to assess unofficial jobs like day-to-day work.

## 5. Conclusion

This study demonstrated the prevalence and effect of different sociodemographic factors and determinants on the parasitic infection, malnutrition, and anemia. The prevalence of parasitic infection among school-age children (6–16 years old) in Ghindae area was high (45.3%). Though there might be many factors for the significantly increased parasitic infection, unsafe drinking water sources from streams and springs are among the core problems along with decreased awareness on personal hygienic and sanitation practices.

Undernutrition is widely prevalent among students in Ghindae area. Height for age and BMI for age scores of all participants were found to be below the 2006 WHO standards. Residency was also found to be an important factor associated with malnutrition. Students of rural areas like Embatkala and Dongolo had two times more odds of having malnutrition. The magnitude of anemia (12.4%) in this study is considered a mild public health problem as WHO recommends iron supplementation for school-aged children, if anemia prevalence exceeds 40%. This research is a beneficial exploratory reference study on risk factors leading to intestinal parasitosis, malnutrition, and anemia in the study area. However, it should be noted that the cross-sectional nature of the study made any inference on causal relationship among variables impossible.

### 5.1. Key Interventional Recommendations

Concerned policy makers should strongly work to increase access and improve sources of clean drinking water in Ghindae area using installation of mass water filters and chlorination of drinking water. Regular programs on increasing awareness and practice of hand washing should be integrated with routine educational activities of the schools. Students living in rural areas of Ghindae require more attention for nutritional interventions. Further studies with more robust methods are required to explore dietary pattern and nutrient intake and their association with nutritional status for better understanding of school-age children malnutrition.

## Figures and Tables

**Table 1:** Prevalence of intestinal parasitosis, malnutrition, and anemia in Ghindae area, Eritrea, 2018 (*n* = 450).

Characteristics	Total	Intestinal parasitosis		Malnutrition		Anemia	
	N (%)	No.	(%)	No.	(%)	No.	(%)
Sex							
Male	215 (47.8)	96	44.7	82	38.1	20	9.3
Female	235 (52.2)	108	46.0	84	35.7	36	15.3
Age groups (in years)							
6–8	133 (29.6)	65	48.9	41	30.8	17	12.8
9–11	146 (32.4)	66	45.2	48	32.9	13	8.9
12–16	171 (38)	73	42.7	77	45.0	26	15.2
Total	450	204	45.3	166	36.9	56	12.4

**Table 2:** Distribution of intestinal parasitosis, malnutrition, and anemia among the six administrative units in Ghindae area, Eritrea, 2018 (*n* = 450).

Administrative units in Ghindae area													
	Dongolo		Debalko		Huxiet		Ferrovia		Mmhdar		Embatkala		*p* value
	No.	(%)	No.	(%)	No.	(%)	No.	(%)	No.	(%)	No.	(%)	
Intest. parasitosis	12	5.9	48	23.5	23	11.3	46	22.5	64	31.4	11	5.4	*p* > 0.05
Protozoan	6	3.5	37	21.5	20	11.6	42	24.4	56	32.6	11	6.4	*p* > 0.05
*E. histolytica/dispar*	5	4.5	26	23.6	14	12.7	27	24.5	33	30	5	4.5	*p* > 0.05
*G. duodenalis*	5	5.2	21	21.6	9	9.3	24	24.7	32	33	6	6.2	*p* > 0.05
Helminthes	7	14.9	15	31.9	6	12.8	6	12.8	13	27.7	0	0	0.042
*H. nana*	3	7	15	34.9	6	14	6	14	13	30.2	0	0	*p* > 0.05
Hookworm	4	80	1	20	0	0	0	0	0	0	0	0	0.001
Multiple infection	4	9.7	14	30.4	5	10.9	10	21.7	13	28.3	0	0	*p* > 0.05
Malnutrition	16	9.6	43	25.9	6	3.6	40	24.1	42	25.3	19	11.4	0.001
Stunting	6	7.2	19	22.6	1	1.2	30	36.1	25	30.1	2	2.4	0.001
Low BMI for age	12	12.6	27	28.4	6	6.3	13	13.7	20	21.1	17	17.9	0.001
Anemia	7	12.5	25	44.6	4	7.1	8	14.3	11	19.6	1	1.8	0.004

**Table 3:** Potential risk factors associated with intestinal parasitic infections in Ghindae area, Eritrea, 2018 (*n* = 450).

Variables	Category	Total *N* (%)	Protozoan		Helminthes	
			*N* (%)	COR	*N* (%)	COR
Gender	Male	215 (47.8)	80 (37.2)	1	22 (10.2)	1
	Female	235 (52.2)	92 (39.1)	1.09 (0.74, 1.59)	25 (10.6)	1.04 (0.570, 1.91)
Age	6–11	279 (62)	108 (38.7)	1	34 (12.2)	1
	12–16	171 (38)	64 (37.4)	0.95 (0.64, 1.40)	13 (7.6)	0.59 (0.30, 1.16)
Ethnic group	Tigrigna	156 (34.7)	50 (32.1)	1	22 (14.1)	1
	Tigre	179 (39.8)	71 (39.7)	1.39 (0.89, 2.19)	14 (7.8)	0.52 (0.26, 1.05)
	Saho	110 (24.4)	50 (45.5)	**1.77 (1.07, 2.93)**	10 (9.1)	0.61 (0.28, 1.34)
	Other	5 (1.1)	1 (20)	0.530 (0.058, 4.87)	1 (20)	1.52 (0.16, 14.26)
Maternal age	0–24	10 (2.3)	4 (40)	1	4 (40)	1
	25–40	343 (77.6)	133 (38.8)	0.95 (0.26, 3.43)	30 (8.7)	**0.14 (0.04, 0.54)**
	>40	89 (20.1)	31 (34.8)	0.80 (0.21, 3.06)	10 (11.2)	**0.19 (0.05, 0.79)**
Occupation of mother	Housewife	354 (80.1)	138 (39)	1	38 (10.7)	1
	Merchant	5 (1.1)	1 (20)	0.39 (0.043, 3.54)	2 (40)	5.54 (0.898, 34.2)
	Government worker	38 (8.6)	12 (31.6)	0.72 (0.35, 1.48)	2 (5.3)	0.46 (0.11, 1.99)
	Farmer	2 (0.5)	0 (0)	0.001	1 (50)	8.31 (0.51, 135.6)
	Other	43 (9.7)	20 (46.5)	1.36 (0.72, 2.57)	3 (7)	0.62 (0.18, 2.11)
Hand hygiene	Clean	286 (63.3)	78 (47.6)	1	24 (14)	1
	Unclean	164 (36.4)	94 (32.9)	**1.85 (1.25, 2.75)**	23 (8.4)	1.78 (0.97, 3.27)
Knowing purpose of washing hands	Yes	129 (28.7)	152 (36.5)	1	43 (10.3)	1
	No	34 (7.6)	20 (58.8)	**2.48 (1.21, 5.05)**	4 (11.8)	1.16 (0.39,3.44)
Water source	River/spring	86 (19.1)	37 (43)	**3.11 (1.29, 7.52)**	8 (9.3)	1
	Pipe	323 (71.8)	127 (39.3)	**2.67 (1.19, 5.97)**	29 (9)	0.96 (0.42, 2.19)
	Water truck	41 (9.1)	8 (19.5)	1	10 (24.4)	3.15 (0.14, 8.71)
History of bloody diarrhea	No	321 (89.4)	128 (39.9)	1	27 (8.4)	1
	Yes	38 (10.6)	14 (36.8)	0.89 (0.44, 1.76)	8 (21.1)	**2.91 (1.21, 6.96)**
Treatment in last month	No	252 (70.8)	92 (36.5)	1	26 (10.3)	1
	Yes	104 (29.2)	48 (46.2)	1.49 (0.94, 2.37)	10 (9.6)	0.93 (0.43, 1.99)
Type of latrine	Modern	145 (37.4)	52 (35.9)	1	14 (9.7)	1
	Traditional	243 (62.6)	99 (40.7)	1.23 (0.80, 1.88)	28 (11.5)	1.21 (0.62, 2.40)
Stool consistency	Formed	183 (40.7)	54 (29.5)	1	18 (9.8)	1
	Soft	219 (48.7)	94 (42.9)	**1.80 (1.19, 2.72)**	24 (11)	1.13 (0.59, 2.15)
	Loose	45 (10)	23 (51.1)	**2.50 (1.28, 4.86)**	5 (11.1)	1.12 (0.40, 3.27)
	Watery	3 (0.7)	1 (33.3)	1.19 (0.11, 13.5)	1 (30)	1.13 (0.75, 1.72)

**Table 4:** Potential risk factors associated with malnutrition, stunting, wasting, and anemia in Ghindae area, Eritrea, 2018 (*n* = 450).

	Category	Total *N* (%)	Malnutrition		Low BMI for age		Low height for age		Anemia	
			*N* (%)	COR	*N* (%)	COR	*N* (%)	COR	*N* (%)	COR
Gender	Male	215 (47.8)	82 (38.1)	1	51 (23.8)	1	35 (16.3)	1	20 (9.3)	1
	Female	235 (52.2)	84 (35.7)	0.90 (0.62, 1.32)	44 (18.8)	0.74 (0.47, 1.17)	48 (20.4)	1.32 (.82, 2.14)	36 (15.3)	1.77 (0.98, 3.15)
Age	6–11	279 (62)	89 (31.9)	1	48 (17.3)	1	43 (15.4)	1	30 (10.8)	1
	12–14	171 (38)	77 (45.0)	**1.75 (1.18, 2.59)**	47 (27.6)	**1.83 (1.16, 2.89)**	40 (23.4)	**1.68 (1.04, 2.71)**	26 (15.2)	1.48 (0.85, 2.62)
Religion	Muslim	297 (66.3)	107 (35.9)	1	57 (19.2)	1	58 (19.5)	1	38 (12.8)	1
	Orthodox	136 (30.4)	56 (40.9)	1.23 (0.82, 1.87)	36 (26.5)	1.52 (0.94, 2.45)	24 (17.5)	0.88 (0.52, 1.48)	17 (12.4)	0.97 (0.53, 1.78)
	Catholic	13 (2.9)	2 (15.4)	0.33 (0.07, 1.49)	1 (7.7)	0.35 (0.05, 2.75)	1 (7.7)	0.35 (0.04, 2.71)	1 (7.7)	0.57 (0.07, 4.51)
	Protestant	2 (0.4)	1 (50)	1.79 (0.11, 28.8)	1 (50)	4.21 (0.26, 68.3)	0 (0)	-	0 (0)	-
Grade	1–3	174 (38.7)	55 (31.6)	1	25 (14.5)	1	32 (18.4)	1	23 (13.2)	1
	4–5	112 (24.9)	36 (32.1)	1.03 (0.62, 1.71)	22 (19.6)	1.45 (0.77, 2.72)	16 (14.3)	0.74 (0.38, 1.42)	11 (9.8)	0.98 (0.52, 1.84)
	6–8	164 (36.4)	75 (45.7)	**1.82 (1.17, 2.84)**	48 (29.4)	**2.47 (1.44, 4.25)**	35 (21.3)	1.20 (0.71, 2.05)	22 (13.4)	0.70 (0.32, 1.51)
Mother occupation	Housewife	354 (80.1)	123 (34.7)	1	69 (19.5)	1	65 (18.4)	1	49 (13.8)	1
	Other	88 (19.9)	40 (45.5)	1.57 (0.98, 2.51)	24 (27.6)	1.57 (0.92, 2.69)	17 (19.3)	1.07 (0.59, 1.93)	6 (6.8)	0.46 (0.19, 1.10)
Residence area	Urban	383 (85.3)	131 (34.2)	1	66 (17.3)	1	75 (19.6)	1	48 (12.5)	1
	Rural	66 (14.7)	35 (53)	**2.17 (1.28, 3.68)**	29 (43.9)	**3.74 (2.15, 6.51)**	8 (12.1)	0.57 (0.26, 1.24)	8 (12.1)	0.96 (0.43, 2.14)
Waste disposal	Improper	106 (23.6)	49 (46.2)	1	31 (29.2)	1	21 (19.8)	1	12 (11.3)	1
	Proper	344 (76.4)	117 (34)	**0.6 (0.38, 0.93)**	64 (18.7)	**0.55 (0.33, 0.91)**	62 (18.0)	0.89 (0.51, 1.54)	44 (12.8)	0.87 (0.44, 1.71)
Parental status	Living together	289 (65.4)	109 (37.7)	1	62 (21.5)	1	54 (18.7)	1	37 (12.8)	1
	Divorce	27(6.1)	14 (51.9)	1.77 (0.81, 3.92)	10 (37.0)	2.15 (0.94, 4.94)	4 (14.8)	0.76 (0.25, 2.28)	2 (7.4)	0.89 (0.46, 1.7)
	Widowed	20 (4.5)	2 (10)	0.18 (0.04, 0.81)	1 (5)	0.19 (0.03, 1.47)	1 (5.0)	0.23 (0.03, 1.75)	1 (5.0)	0.48 (0.10, 2.26)
	Separated	106 (24)	39 (36.8)	0.96 (0.61, 1.52)	22 (21.2)	0.98 (0.57, 1.70)	22 (20.8)	1.14 (0.65, 1.98)	15 (14.2)	0.32 (0.04, 2.56)

**Table 5:** Multivariate logistic regression analysis showing predictors of malnutrition and anemia among school children, *n* = 450.

Variable	Category	Malnutrition		Anemia	
		AOR (95% CI)	*p* value	AOR (95% CI)	*p* value
Sex	Male	1	0.91	1	0.09
	Female	1.03 (0.65, 1.61)		1.76 (0.91, 3.42)	
Age	6–11	1	0.41	1	0.035
	12–16	1.38 (0.64, 2.94)		**1.36 (1.09, 3.42)**	
Grade	Elementary	1	0.23	1	0.15
	Junior	1.60 (0.75, 3.43)		0.44 (0.14, 1.37)	
Residence area	Urban	1	0.037	1	0.42
	Rural	**2.03 (1.04, 3.96)**		0.65 (0.23, 1.84)	
House head monthly salary	0–500	1.18 (0.45, 3.03)	0.73	1.94 (.22, 17.1)	0.55
	501–1500	1.22 (0.50, 2.97)	0.64	4.98 (.63, 38.8)	0.12
	1501–2500	0.71 (0.24, 2.09)	0.53	5.61 (0.64, 48.6)	0.11
	Above 2500	1	0.54	1	
Parental status	Living together	1		1	0.62
	Divorce	1.52 (0.59, 3.90)	0.37	0.33 (0.04, 2.69)	0.30
	Widowed	0.15 (0.018, 1.19)	0.07	0.41 (0.04, 3.46)	0.41
	Separated	0.95 (0.55, 1.63)	0.86	1.04 (0.49, 2.19)	0.91
Waste disposal	Improper	1	0.52	1	0.65
	Proper	0.83 (0.47, 1.48)		0.82 (0.36, 1.88)	

**Table 6:** Multivariate logistic regression analysis showing predictors of intestinal protozoan infection among school children, *n* = 450.

Intestinal protozoan infection			
Category	COR (95% CI)	AOR (95% CI)	*p* value
Gender			
Male	1	1	0.249
Female	1.09 (0.74, 1.59)	1.27 (0.85, 1.93)	

Age			
6–11	1	1	0.986
12–16	0.95 (0.64, 1.40)	1.00 (0.65, 1.53)	

Ethnic group			
Tigrigna	1	1	0.346
Tigre	1.39 (0.89, 2.19)	1.17 (0.73, 1.89)	0.501
Saho	**1.77 (1.07, 2.93)**	1.55 (0.89, 2.70)	0.116
Other	0.530 (0.058, 4.87)	0.40 (0.04, 3.95)	0.433

Knowing purpose of washing hands			
Yes	1	1	0.035
No	**2.48 (1.21, 5.05)**	**2.23 (1.056, 4.71)**	

Hand hygiene			
Clean	1	1	0.004
Unclean	**1.85 (1.25, 2.75)**	**1.89 (1.23, 2.90)**	

Water source			
River/spring	**3.11 (1.29, 7.52)**	**2.41 (1.04, 5.58)**	0.038
Pipe	**2.67 (1.19, 5.97)**	2.30 (0.91, 5.85)	0.078
Water truck	1	1	

Stool consistency			
Formed	1	1	0.010
Soft	**1.80 (1.19, 2.72)**	1.92 (1.25, 2.97)	0.003
Loose	**2.50 (1.28, 4.86)**	2.43 (1.22, 4.85)	0.012
Watery	1.19 (0.11, 13.5)	1.13 (0.095, 3.44)	0.922

## Data Availability

The data used to support the findings of this study are available from the corresponding author upon reasonable request.

## Abbreviations

BMI: Body mass index
BAZ: BMI for age Z-score
HAZ: Height for age Z-score
IPI: Intestinal parasitic infection
STH: Soil-transmitted helminth
WHO: World Health Organization
Hb: Hemoglobin.
